# Chest CT Images for COVID-19: Radiologists and Computer-Based Detection

**DOI:** 10.3389/fmolb.2021.614207

**Published:** 2021-03-30

**Authors:** Qingli Dou, Jiangping Liu, Wenwu Zhang, Yanan Gu, Wan-Ting Hsu, Kuan-Ching Ho, Hoi Sin Tong, Wing Yan Yu, Chien-Chang Lee

**Affiliations:** ^1^Department of Emergency Medicine, The Second Affiliated Hospital of Shenzhen University, Shenzhen, China; ^2^Department of Epidemiology, Harvard T.H. Chan School of Public Health, Boston, MA, United States; ^3^Radiology Department, St George Hospital Sydney, Kogarah, NSW, Australia; ^4^Li Ka Shing Faculty of Medicine, The University of Hong Kong, Pok Fu Lam, Hong Kong; ^5^Department of Emergency Medicine, National Taiwan University Hospital, Taipei, Taiwan

**Keywords:** COVID-19, 2019-nCoV, chest computed tomography, computer-aided detection, computer-based detection

## Abstract

**Background:**

Characteristic chest computed tomography (CT) manifestation of 2019 novel coronavirus (COVID-19) was added as a diagnostic criterion in the Chinese National COVID-19 management guideline. Whether the characteristic findings of Chest CT could differentiate confirmed COVID-19 cases from other positive nucleic acid test (NAT)-negative patients has not been rigorously evaluated.

**Purpose:**

We aim to test whether chest CT manifestation of 2019 novel coronavirus (COVID-19) can be differentiated by a radiologist or a computer-based CT image analysis system.

**Methods:**

We conducted a retrospective case-control study that included 52 laboratory-confirmed COVID-19 patients and 80 non-COVID-19 viral pneumonia patients between 20 December, 2019 and 10 February, 2020. The chest CT images were evaluated by radiologists in a double blind fashion. A computer-based image analysis system (uAI System, Lianying Inc., Shanghai, China) detected the lesions in 18 lung segments defined by Boyden classification system and calculated the infected volume in each segment. The number and volume of lesions detected by radiologist and computer system was compared with Chi-square test or Mann-Whitney *U* test as appropriate.

**Results:**

The main CT manifestations of COVID-19 were multi-lobar/segmental peripheral ground-glass opacities and patchy air space infiltrates. The case and control groups were similar in demographics, comorbidity, and clinical manifestations. There was no significant difference in eight radiologist identified CT image features between the two groups of patients. There was also no difference in the absolute and relative volume of infected regions in each lung segment.

**Conclusion:**

We documented the non-differentiating nature of initial chest CT image between COVID-19 and other viral pneumonia with suspected symptoms. Our results do not support CT findings replacing microbiological diagnosis as a critical criterion for COVID-19 diagnosis. Our findings may prompt re-evaluation of isolated patients without laboratory confirmation.

## Introduction

Due to high transmissibility and so far lack of proven treatment, the 2019 novel coronavirus disease, 2019-nCoV, has quickly disseminated worldwide (Bogoch et al., 2020; Dong et al., 2020). As symptoms of COVID-19 are similar to other acute respiratory infections, diagnosis relies on positive nucleic acid test (NAT). Given the long turnaround time and suboptimal sensitivity of NAT, chest computed tomography (CT) was proposed as a first line diagnostic tool by the Chinese national guideline (trial version 5) (Ai et al., 2020). This however, created problems, particularly resulting isolating patients with COVID-19 with patients with similar respiratory symptoms due to other infections, as well as delaying appropriate treatment for other treatable infections. We believe findings on CT might not be specific enough to differentiate infection from COVID-19 from non-COVID-19 etiology. It is not a coincidence that after the new definition implementation, 14,840 new cases with 242 deaths were reported on February 13th, which was the record by far reported in a single day since the outbreak (Coronavirus COVID-19 Global Cases by John Hopkins, 2020).

Several case series have reported characteristic CT findings of COVID-19, including ground glass opacities in bilateral peripheral lung, crazy-paving changes, reticular thickening, or consolidations (Fang et al., 2020b; Lei et al., 2020). Given that clinically it is difficult to distinguish COVID-19 from other infective causes for patients with respiratory symptoms, we aim to evaluate whether radiologists or a computer-based image analysis system can reliably differentiate COVID-19 cases from non-COVID-19 but suspected patients.

## Materials and Methods

### Study Population

From 20 December, 2019 to 10 February, 2020, 52 laboratory-confirmed COVID-19 patients in Shenzhen were identified that fulfilled the diagnostic criteria of Chinese national guideline (Lin and Li, 2020). The criteria for a confirmed case include documented laboratory evidence, compatible clinical symptoms, and exposure history. Documented laboratory evidence is defined by positive NAT result either from respiratory tract, bronchoalveolar lavage fluid, or blood sample. Compatible clinical symptoms refer to fever, cough, imaging characteristics of pneumonia, and/or normal or decreased white blood cells count or decreased lymphocyte count. Exposure history includes travel/residence history in Wuhan city, contact history with laboratory-confirmed patients, or contact history with patients with fever or respiratory symptoms from Wuhan and its surrounding areas or endemic communities, within 14 days before the onset of illness. We randomly selected 80 laboratory-confirmed non-COVID-19 viral pneumonia patients as controls. These presented with suspected symptoms and exposure history and underwent NAT and chest CT exams in the same period. To be eligible for inclusion as a control, patients must have at least two negative NAT results from two respiratory specimens and laboratory evidence of other respiratory viruses including positive antigen or NAT test results for influenza A, influenza B, parainfluenza, respiratory syntactical virus, or adenovirus.

### Chest CT Evaluation by Radiologists

All CT images were independently retrospectively analyzed using a structured form by two experienced radiologists in a double blinded fashion without knowing the clinical diagnosis. A third senior radiologist was consulted to solve any discrepancy by consensus. Evaluation was focused on the presence of ground glass opacities, patchy infiltration, patchy consolidation, pleural effusion, mediastinal lymphadenopathy, air bronchogram, pleural thickening, and interstitial change.

### Machine-Learning Based CT Lesion Detection and Quantification System

We used the uAI image analysis system (Lianying Intelligent Medical Technology Co., Ltd., Shanghai, China) for detection and quantification of chest CT lesions (Computer-based detection in Supplementary Appendix). The system classifies the lung fields into five lung lobes and 18 lung segments based on Boyden classification, detects infected regions in each anatomical region, and quantifies the cumulative and relative infected volume. The infected volumes between the two groups were also compared in four different CT windows. Different CT windows refer to different status of brightness and contrast of a CT image manipulated via the CT numbers in order to highlight particular structures. This study was approved by the Institutional Review Board of the Second Affiliated Hospital of Shenzhen University.

### Statistical Analysis

Categorical variables were expressed as number and proportion and compared with a Chi-square test. Continuous variables were presented with mean ± standard deviation for data with a normal distribution and tested by Student *t*-test. Data with non-normal distribution were presented with median with interquartile range and compared with independent sample Mann-Whitney *U* test. All tests in this study were two-sided, and *P* < 0.05 was deemed statistically significant. Data was analyzed using SPSS 23.0 software.

## Results

### Study Patients

Of the 52 included case patients, 32 (61.54%) were male with a mean age of 45.61 ± 14.19 years. The duration of the disease course ranged from 1 to 10 days, with a mean of 5.61 ± 2.19 days. Chest CT examination was performed within 10 days of disease onset. Among patients with COVID-19, fever, fatigue, and dry cough were the major presenting symptoms; hypertension, chronic heart failure, and diabetes were the leading comorbidities, and lymphopenia with elevated CRP or lactate levels were the main laboratory findings. There was no significant difference between case and control in demographic, disease course, clinical symptoms, and laboratory findings (Table 1).

**TABLE 1:** Patients characteristics between case and control patients [*n*(%)].

Characteristic	COVID-19 (*N* = 52)	Non-COVID-19 viral pneumonia (*N* = 80)	*P*-value
Age	45.61 ± 14.19	48.69 ± 14.62	0.12
**Sex**			
Female	32 (61.54%)	58 (72.50%)	0.44
Male	20 (38.46%)	22 (27.50%)	0.73
Course of disease	5.61 ± 2.19	8.32 ± 1.82	0.61
Onset of symptom to CT examination	4.32 ± 0.82	6.87 ± 1.89	0.06
**Symptoms**			
Fever	47 (90.38)	71 (88.75)	0.24
Fatigue	44 (84.61)	68 (85.00)	0.08
Dry cough	31 (59.62)	48 (60.00)	0.16
Dyspnea	15 (28.85)	27 (33.75)	0.67
Sore throat	28 (53.85)	49 (61.25)	0.48
**Comorbidities**			
Hypertension	24 (46.15)	39 (48.75)	0.89
Chronic heart failure	10 (19.23)	17 (21.25)	0.85
Diabetes	14 (26.92)	20 (25.00)	0.52
Cerebrovascular disease	9 (17.31)	19 (23.75)	0.37
COPD	11 (21.15)	16 (20.00)	0.94
Chronic kidney disease	4 (7.69)	8 (10.00)	0.30
**Laboratory results**			
White blood cell count (10^9^/L)	13.60 ± 5.38	11.35 ± 5.88	0.39
Neutrophil percentage (%)	20.43 ± 5.86	18.20 ± 5.05	0.59
Lymphocyte percentage (%)	82.45 ± 14.54	85.69 ± 10.08	0.20
C-reactive protein (mg/dL)	93.20 ± 24.01	106.59 ± 29.29	0.10
Lactate (mmol/L)	2.71 ± 1.38	2.58 ± 1.20	0.16

### Comparison of CT Interpretation by Radiologists

In both COVID-19 and non-COVID-19 viral pneumonia patients, ground-glass opacity (Supplementary Figures 1A,B) and patchy airspace infiltrates (Supplementary Figures 2A,B) were the major findings. The lesions could be found in multiple lobes or segments, more often bilateral. There was no statistical significance between the distribution of lesions identified by radiologists in the two groups. The majority of both categories of patients have ground glass opacities and patchy infiltration, followed by patchy consolidation. There is no statistically significant difference in any of the CT manifestations between the two groups (Table 2).

**TABLE 2:** Radiologist interpretation of chest CT before NAT results [*n*(%)].

	COVID-19 (*N* = 52)	Non-COVID-19 viral pneumonia (*N* = 80)	*P-*value
Ground glass opacities	38 (73.07)	57 (71.25)	0.27
Patchy infiltration	30 (57.69)	48 (60.00)	0.08
Patchy consolidation	15 (28.84)	28 (35.00)	0.15
Pleural effusion	4 (7.69)	9 (11.25)	0.54
Mediastinal lymphadenopathy	3 (5.78)	5 (6.25)	0.71
Air bronchogram	11 (21.15)	15 (18.75)	0.19
Pleural thickening	4 (7.69)	6 (7.50)	0.57
Interstitial change	10 (19.23)	16 (20.00)	0.29

### Comparison of Computer System Detected Infected Lung Volume

Compared between COVID-19 and non-COVID-19 viral pneumonia patients, there was no significant difference in the computer system detected infected lung volume/percentage in 5 lung lobes and in different lung segments (Table 3). Similarly, there was no significant difference in the absolute or relative infected lung volume between the two groups of patients (Supplementary Table 1).

**TABLE 3:** Difference in lesions distribution between COVID-19 and non-COVID-19 viral pneumonia patients.

Affected Lung field	COVID-19		Non-COVID-19 viral pneumonia		*P*^*a*^	*P*^*b*^
	Infection volume (cm^3^)	Percentage of infection (%)	Infection volume (cm^3^)	Percentage of infection (%)		
Right upper lobe	29.55 ± 10.41	3.93 ± 1.11	40.76 ± 13.24	11.66 ± 3.67	0.70	0.71
Right middle lobe	11.61 ± 6.64	0.63 ± 0.20	25.35 ± 7.51	4.57 ± 1.90	0.22	0.20
Right lower lobe	45.93 ± 18.40	7.19 ± 3.41	80.61 ± 41.47	15.56 ± 7.38	0.37	0.93
Left upper lobe	22.17 ± 8.54	1.19 ± 0.11	34.60 ± 10.41	8.67 ± 2.00	0.90	0.79
Left lower lobe	48.42 ± 22.98	8.67 ± 1.45	105.76 ± 32.03	22.15 ± 8.02	0.52	0.75

*^*a*^Comparison of the infection volume in COVID-19 group and non-COVID-19 viral pneumonia. ^*b*^Comparison of the infection ratio of the affected sites in COVID-19 group and non-COVID-19 viral pneumonia group.*

### Sensitivity Analysis Under Different Radiodensity Ranges for Machine Detection

The lesions’ infection volume/percentage for COVID-19 and non-COVID-19 patients were stratified by four different CT number ranges (Supplementary Table 2). Lesions in the (−750, −300) Hounsfield units (HU) range were arbitrarily chosen to reflect ground-glass opacity, while those with CT numbers in the (−300, 50) range were considered as denser airspace infiltrates and consolidation. Anything less than −750 becomes harder to differentiate from normal lung tissue. An extreme number 50 was chosen, beyond which infection becomes unlikely, though not impossible, as the number starts to get into the soft tissue mass range. No statistically significant difference in the infected volumes between the two groups was found in any of the four ranges.

## Discussion

In our case-control study, we selected controls in the real world settings, used both radiologist and computer system to evaluate the CT image difference between two groups of patients. We confirmed the main CT manifestations of patients with COVID-19 were multi-lobar/segmental peripheral ground-glass opacities and patchy airspace infiltrates. There was no significant difference between the two groups in radiologists’ interpretation and the volume of software system detected lesions.

The Severe Acute Respiratory Syndrome Coronavirus-2 (SARS-CoV-2) primarily invades the lung parenchyma (Lin and Li, 2020). Because of the long turnaround time and suboptimal sensitivity of NAT for SARS-CoV-2, whether chest CT can be used as a first line diagnostic rule to differentiate COVID-19 from other viral pneumonia is of critical importance in both clinical and public health perspectives. Chen et al. (2020) studied 29 patients with COVID-19 showed that the chest image lesions were mostly bilateral and multiple with patchy shadows and ground glass opacities (Chan et al., 2020; Fang et al., 2020a). Pan et al., reported CT images of COVID-19 are diverse in the early stages, which may present ground-glass opacities, pulmonary consolidation and nodules (Pan and Guan, 2020). These studies were case series without suitable control groups (Chen et al., 2020; Fang et al., 2020b; Kanne, 2020; Lei et al., 2020; Pan and Guan, 2020). We found Radiologists’ interpretation alone or computer-based lesion detection cannot differentiate COVID-19 form other viral pneumonia. Such findings were robust under different anatomic sites or CT density ranges.

There are pros and cons using clinical diagnosis as a case definition. Clinical diagnosis allows early isolation with initial false negative NAT, slows transmission, and implements treatment early. However, the use of chest CT as a first line diagnostic method may miss early/mild disease, promote cross-infection in the CT room, increase radiation exposure, and consume enormous resources of disinfection.

Our findings have multiple implications. First, clinicians should not rely on initial chest CT findings to diagnose COVID-19 in the absence of laboratory confirmation. Second, patients who had been diagnosed with COVID-19 based on the clinical grounds should be re-evaluated with serum antibody tests. Third, patients who were isolated and cohorted with laboratory-confirmed cases in the temporary COVID-19 hospitals of Hubei province may need to be reevaluated aiming for further laboratory evidence including repeated NAT or serum antibody test.

Results of study should be interpreted in light of its shortfalls. Due to the constraints in time and patient number, we could not perform independent validation with an external sample. Comparison between radiologists and Artificial intelligence interpretations was not made as this was not within our study scope, but could be a point of interest for future study. We did not compare serial imaging changes. We used a commercial image analysis system based on deep learning. The system does not provide flexibility in adjustment of machine-learning model or hyperparameters selection. We do not exclude future tailor trained machine learning systems that can differentiate chest CT of COVID-19 from other suspected patients. Lastly, the sample size we collected could provide a mere statistical power of 70% to differentiate a 20% difference between two proportions. The calculation of the power is based on the *Z*-test with a statistical significance level of 0.0.5 and a sample size of 130 patients.

## Conclusion

We documented the non-differentiating nature of initial chest CT between COVID-19 and other viral pneumonia with suspected symptoms. Our results do not support CT findings replacing microbiological diagnosis as a critical criterion for COVID-19 diagnosis.

## Data Availability Statement

The raw data supporting the conclusions of this article will be made available by the authors, without undue reservation.

## Ethics Statement

The studies involving human participants were reviewed and approved by the Institutional Review Board of the Second Affiliated Hospital of Shenzhen University. The patients/participants provided their written informed consent to participate in this study. Written informed consent was obtained from the individual(s) for the publication of any potentially identifiable images or data included in this article.

## Author Contributions

QD and JL: conceptualization, data curation, and formal analysis. QD, JL, WZ, and YG: methodology. W-TH, HT, and WY: project administration. QD, JL, and C-CL: resources. QD and C-CL: supervision. QD, JL, WZ, YG, and C-CL: writing – original draft. QD, JL, W-TH, K-CH, HT, WY, and C-CL: writing – review and editing. All authors contributed to the article and approved the submitted version.

## Conflict of Interest

The authors declare that the research was conducted in the absence of any commercial or financial relationships that could be construed as a potential conflict of interest.
