# Supplementary material for: Chest CT Images for COVID-19: Radiologists and Computer-Based Detection
Source: Front Mol Biosci. 2021 Mar 30;8:614207. doi: 10.3389/fmolb.2021.614207 (PMC8044917; doi:10.3389/fmolb.2021.614207)
Supplement: Supplementary file 1 [file Data_Sheet_1.PDF]

Appendix

Chest CT Images for COVID-19: Radiologists and Computer-based detection

Table of Contents

Table S1. Difference in the distribution of affected lung segments between COVID-19 and non-COVID-19 viral pneumonia patients

Table S2. Difference in the distribution of CT HU values between COVID-19 and non-COVID-19 viral pneumonia patients

Supplemental Figures 1. Chest CT image of 57-year-old COVID patient shows scattered ground-glass opacity in right lower lung with irregular shape and unclear borer (A). The uAI system could automatically detect the lesion and marked it for visualization (B).

Supplemental Figures 2. The chest CT image of a 62-year-old virus pneumonia of non-COVID-19 viral pneumonia patient shows bilateral scattered patch infiltrates with heterogeneous lesion density and blurred edges. The distribution was mainly subpleural, along the blood vessels with air bronchogram in some lesions (A). The uAI system can capture the lesion for quantitative analysis (B).

Online supplement. Computer-based detection

Table S1. Difference in the distribution of affected lung segments between COVID-19 and non-COVID-19 viral pneumonia patients

Affected lung segment	COVID-19		non-COVID-19 viral pneumonia			
	Infection volume(cm^3)	Percentage of infection(%)	Infection volume(cm^3)	Percentage of infection(%)	P^a	P^b
Left upper lobe						
$B_{1/2}$	0.50±0.21	0.14±0.06	6.94±1.94	4.41±0.98	0.55	0.61
B_3	0.38±0.12	0.09±0.03	5.56±1.18	4.09±1.05	0.14	0.06
B_4	1.17±0.36	0.30±0.09	1.96±0.49	0.96±0.28	0.29	0.33
B_5	1.25±0.54	0.66±0.29	15.50±4.37	8.53±2.63	0.19	0.16
Left lower lobe						
B_6	15.89±6.12	6.35±2.36	41.85±3.77	21.53±6.07	0.36	0.06
B_8	4.26±1.52	1.34±0.52	30.88±6.65	13.59±4.44	0.77	0.88
B_9	9.72±3.19	4.01±1.31	46.29±13.61	27.69±10.87	0.81	0.52
B_{10}	12.91±5.57	6.68±2.61	26.79±8.00	19.66±7.09	0.95	0.61
Right upper lobe						
B_1	1.54±0.62	0.42±0.18	12.82±4.78	5.82±2.24	0.71	0.77
B_2	24.21±9.12	16.69±5.57	8.43±3.38	10.99±4.19	0.60	0.48
B_3	22.48±7.16	6.47±2.08	7.19±4.08	5.38±1.22	0.70	0.84
Right middle lobe						
B_4	1.16±0.56	0.48±0.24	5.32±1.61	3.35±1.41	0.90	0.50
B_5	0.27±0.10	0.06±0.02	8.24±2.42	4.69±1.37	0.56	0.43
Right lower lobe						
B_6	20.72±14.02	20.33±10.67	20.83±8.69	17.95±7.08	0.94	0.90
B_7	1.37±0.86	1.37±0.83	6.02±1.53	15.51±3.48	0.73	0.83
B_8	4.12±1.59	1.35±0.01	26.95±9.41	10.28±4.64	0.40	0.18
B_9	8.99±5.75	6.14±3.54	18.71±10.69	16.16±6.54	0.59	0.82
B_{10}	31.44±27.14	28.23±14.82	26.49±11.15	21.70±11.26	0.21	0.76

Note: Classification of lung segments based on the Boyden classification of bronchi[11].Left upper lobe: apicoposterior segment = B1/2; anterior segment = B3; superior lingular segment = B4;

inferior lingular segment = B5. Left lower lobe: superior segment = B6; anteromedial segment = B8; lateral segment = B9; posterior segment = B10. Right upper lobe: apical segment = B1; posterior segment = B2; anterior segment = B3. Right middle lobe: lateral segment = B4; medial segment = B5. Right lower lobe: superior segment = B6; medial segment = B7; anterior segment = B8; lateral segment = B9; posterior segment = B10.a: Comparison of the infection volume of the affected lung segment between COVID-19 group and non-COVID-19 viral pneumonia group; b: Comparison of the infection ratio of the affected lung segment between COVID-19 group and non-COVID-19 viral pneumonia group.

Table S2. Difference in the distribution of CT HU values between COVID-19 and non-COVID-19 viral pneumonia patients

CT number (HU)	COVID-19		non-COVID-19 viral pneumonia			
	Infection volume(cm^3)	Percentage of infection(%)	Infection volume(cm^3)	Percentage of infection(%)	P^a	P^b
(,-750)	12.05±6.13	4.47±7.41	19.13±8.65	7.74±2.02	0.68	0.47
[-750,-300)	59.30±37.94	64.62±16.40	81.24±38.74	58.11±22.33	0.52	0.16
[-300,50)	29.72±19.42	15.85±13.13	61.10±27.07	21.82±9.75	0.68	0.06
[50,)	12.14±11.01	15.06±11.11	22.05±11.88	12.33±3.80	0.24	0.23

Note: a: Comparison of the infection volume in different CT number ranges between COVID-19 group and non-COVID-19 viral pneumonia group; b: Comparison of the infection ratio of different CT number ranges between COVID-19 group and non-COVID-19 viral pneumonia group.

Supplemental Figures 1. Chest CT image of 57-year-old COVID patient shows scattered ground-glass opacity in right lower lung with irregular shape and unclear borer (A). The uAI system could automatically detect the lesion and marked it for visualization (B).

A B

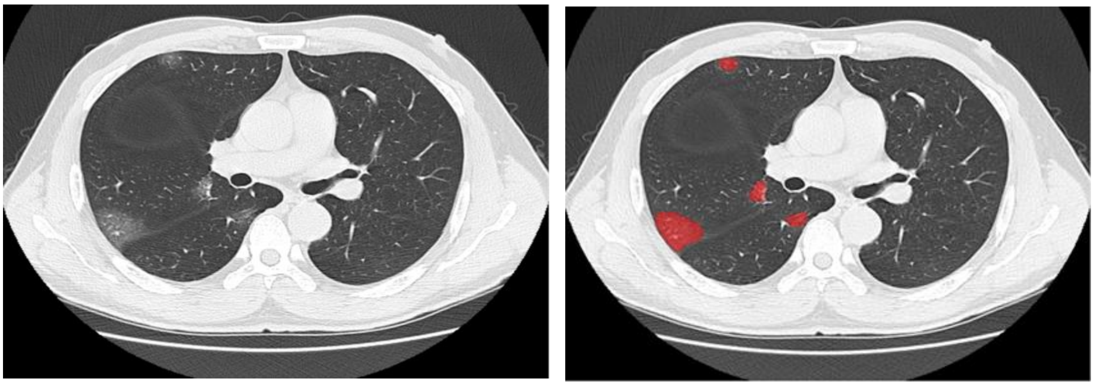

Supplemental Figures 2. The chest CT image of a 62-year-old virus pneumonia of non-COVID-19 viral pneumonia patient shows bilateral scattered patch infiltrates with heterogeneous lesion density and blurred edges. The distribution was mainly subpleural, along the blood vessels with air bronchogram in some lesions (A). The uAI system can capture the lesion for quantitative analysis (B).

A B

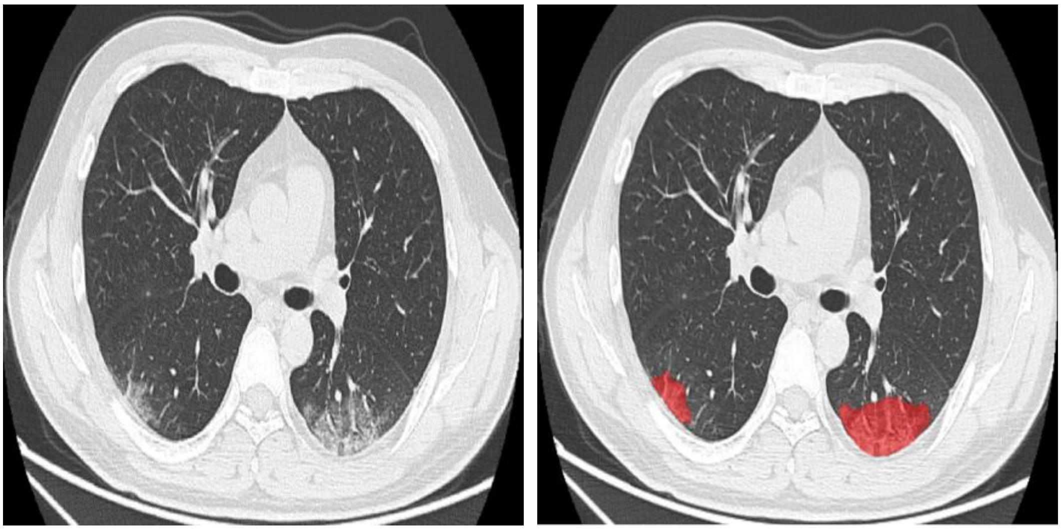

Computer-based detection

The system was developed based on three component systems including multi-scale detection convolutional neural network, cascade deep learning algorithm, and end-to-end organ segmentation network. The multi-scale detection neural network recovers multi-scale feature information through up-sampling in the neural network, and use skip layer connections to fuse fine-scale and coarse-scale features, thereby enhancing the accuracy of feature space and improving the detection sensitivity. The cascaded deep learning detection network connected different detection networks in series. Each layer of detection network is responsible for distinguishing some suspected pneumonia areas from real pneumonia areas, solving the problem of imbalance of positive and negative samples during network training, and achieving complete differentiation between non-lesion areas and lesion areas. The end-to-end organ segmentation network performs fast positioning through coarse-scale networks and performed segmentation in the target small-scale area on the fine-scale network, thereby achieving accurate segmentation of lung regions and lung segments while significantly reducing memory consumption.
